# Evanescent Wave Optical-Fiber Aptasensor for Rapid Detection of Zearalenone in Corn with Unprecedented Sensitivity

**DOI:** 10.3390/bios12070438

**Published:** 2022-06-22

**Authors:** Haixu Zhao, Shang Ren, Zhenzhe Wei, Xinhui Lou

**Affiliations:** Department of Chemistry, Capital Normal University, Xisanhuan North Road. 105, Beijing 100048, China; 2200702061@cnu.edu.cn (H.Z.); 2200702033@cnu.edu.cn (S.R.); 2190702031@cnu.edu.cn (Z.W.)

**Keywords:** zearalenone, evanescent wave optical-fiber aptasensor, aptamer, mycotoxin

## Abstract

Zearalenone (ZEN) is a common mycotoxin pollutant found in agricultural products. Aptamers are attractive recognition biomolecules for the development of mycotoxin biosensors. Even though numerous aptasensors have been reported for the detection of ZEN in recent years, many of them suffer from problems including low sensitivity, low specificity, tedious experimental steps, high-cost, and difficulty of automation. We report here the first evanescent wave optical-fiber aptasensor for the detection of ZEN with unprecedented sensitivity, high specificity, low cost, and easy of automation. In our aptasensor, a 40-nt ZEN-specific aptamer (8Z_31_) is covalently immobilized on the fiber. The 17-nt fluorophore Cy5.5-labeled complementary DNA strand and ZEN competitively bind with the aptamer immobilized on the fiber, enabling the signal-off fluorescent detection of ZEN. The coating of Tween 80 enhanced both the sensitivity and the reproducibility of the aptasensor. The sensor was able to detect ZEN spiked-in the corn flour extract with a semilog linear detection range of 10 pM-10 nM and a limit of detection (LOD, S/N = 3) of 18.4 ± 4.0 pM (equivalent to 29.3 ± 6.4 ng/kg). The LOD is more than 1000-fold lower than the maximum ZEN residue limits set by China (60 μg/kg) and EU (20 μg/kg). The sensor also has extremely high specificity and showed negligible cross-reactivity to other common mycotoxins. In addition, the sensor was able to be regenerated for 28 times, further decreasing its cost. Our sensor holds great potential for practical applications according to its multiple compelling features.

## 1. Introduction

Zearalenone (ZEN) is one of the most widely distributed mycotoxins produced by Fusarium [1]. It is a phenolic isophthalic acid lactone, and its molecular structure is similar to that of estrogen (estradiol), so ZEN has estrogen-like activity [2]. It can cause estrogenic effects and can also enter humans and animals through food and feed, resulting in reproductive disorders and a potential threat to mammals, which may even lead to death [3,4,5]. The main pollution sources of ZEN are grains, including major crops such as corn, wheat, and sorghum, as well as milk and spices [6,7,8]. ZEN contaminates 25% of the world’s food [9] and many countries have limited the content of ZEN in variety of crops and food [10]. For examples, the maximum residue limits for ZEN in corn are 60 [11] and 20 μg/kg [12] in China and EU, respectively. Therefore, there is an urgent need to establish an easy-to-operate, sensitive, low-cost, and fast method to detect ZEN in crops to protect the health and safety of humans and animals.

The chromatography and mass spectrometry-based methods are the standard methods routinely used in laboratories and have high sensitivity and accuracy. However, these methods require complex sample preparation steps, high cost, and professional personnel, therefore they are not capable of on-site applications. Antibody-based methods such as ELISA and lateral flow test strips have developed into commercialized products but suffer from the great batch-to-batch variation of antibodies. Different from antibodies, nucleic acid aptamers are in vitro isolated binding ligands [13,14]. Aptamers have the excellent batch-to-batch consistency since they are prepared by organic synthesis instead of in vivo immunization reaction as used for the preparation of antibodies. Aptamers also have other advantages over antibodies including high stability, low cost, easy chemical modification and probe design, and great flexibility to be compatible with various detection platforms. With the maturing of the in vitro aptamer selection technologies (SELEX) [15,16,17,18,19,20], the development of aptasensors for the rapid and facile detection of ZEN has become a research hotspot [21].

Over the past ten years, the ZEN-binding aptamers with nanomolar affinity have been isolated by several research groups (Supporting Information (SI) Table S1). Based on these aptamers, different types of ZEN-aptasensors including colorimetric, fluorescence, and electrochemical sensing platforms have been reported (SI Table S2) [22,23]. By using various types of nanomaterials, the sensitivities of the aptasensors have been improved orders of magnitude, especially for the electrochemical aptasensors, which are inherent much more sensitive than optical sensors. However, each aptasensor still suffers from different problems such as low sensitivity, complicated preparation of nanomaterial–biomolecule complexes and electrodes, the need for enzymes, high cost, a long assay time, and so on (Table 1). To overcome these problems, tedious and expensive signal amplification steps and pre-samplings are included, which trades-off the desired benefits of biosensors in cost and assay time.

Evanescent wave optical-fiber sensor (EWOF) is a type of portable device based on the evanescent wave generated on the fiber surface when the total reflection of laser inside the optical fiber [29,30,31]. It has the advantages of miniaturization, automation, and low cost, but suffers from low sensitivity when small molecule-binding aptamers are used as recognition ligands. Due to this limitation, the application of EWOF for the detection of mycotoxins, which typically have low contents within a complex food matrix, is limited [32,33]. Recently, we developed an ultrasensitive evanescent wave optical fiber aptasensor for the detection of small molecules by constructing nanoscale affinity double layer on the fiber (NADL) [34]. The NADL consists of an aptamer layer and a microextraction layer, which can achieve in situ target purification, detection and in situ target enrichment. We demonstrated the ultrasensitive detection of alternariol spiked in the wheat flour [35]. The application of EWOF aptasensor for the detection of ZEN has never been achieved. 

In this work, we report the first EWOF aptasensor for the ultrasensitive, simple, low-cost, and rapid detection of ZEN. For fair comparison with the reported aptasensors (Table 1), we fabricated the NADL-based sensor using the most widely utilized 40-nt ZEN-binding aptamer, 8Z_31_ (SI, Table S1) [36]. The Cy5.5-labeled complementary DNA strand and ZEN competitively bind with the aptamer functionalized on the fiber, enabling signal-off fluorescence detection of ZEN. The coating of Tween 80 greatly enhanced both the sensitivity and the reproducibility of the aptasensor. We evaluated the sensor in terms of sensitivity, specificity, resistance to matrix interference, and regeneration ability. Our sensor shows the unprecedented sensitivity and excellent anti-matrix interference capability. The direct detection of ZEN spiked in the corn flour was achieved simply by diluting the extract within about 1 h total assay time including sample extraction. The sensitivity of our sensor was more than 1000 times lower than the maximum residues limits of ZEN in food samples. The sensor also has excellent specificity, showing negligible cross-reactivity with nine other common mycotoxins. In addition, our sensor can be easily regenerated, which is of great significance to further reduce the cost. The estimated cost per test is much lower than the most used ELISA kits and test strips. Our sensor has great potential for practical applications and the same technique can be extended to fabricate aptasensors for the ultrasensitive detection of other mycotoxins. 

## 2. Materials and Methods

### 2.1. Materials

The amino group and spacer modified ZEN binding-aptamer 8Z_31_ (5′-NH_2_-AAAAAAAAAATCATCTATCTATGGTACATTACTATCTGTAATGTGATATG-3′) and the fluorescent dye labeled cDNA (5′-CATTACAGATAGTAA TG-Cy5.5-3′) were synthesized and purified through high performance liquid chromatography (HPLC) by Sangon Biotech (Shanghai, China). All the DNA probe stock solutions at 100 μM were prepared in RNase free water and stored at −80 °C. The binding buffer contains 100 mM NaCl, 20 mM Tris–HCl, 2 mM MgCl_2_, 5 mM KCl, and 1 mM CaCl_2_ (pH 7.4). Tris (hydroxymethyl) methyl aminomethane (Tris), sodium chloride (NaCl), magnesium chloride (MgCl_2_), potassium chloride (KCl), and calcium chloride (CaCl_2_) were all purchased from the Shanghai Sangon Biotechnology Co., Ltd. (Shanghai, China). Sulphuric acid (H_2_SO_4_), hydrochloric acid (HCl), and toluene were all purchased from Beijing Chemical Works (Beijing, China). Vomitoxin (DON), ochratoxin (OTA), aflatoxin B1 (AFB1), aflatoxin B2 (AFB2), aflatoxin G1 (AFG1), aflatoxin G2 (AFG2), aflatoxin M1 (AFM1), fumonisin B1 (FB1), and fumonisin B2 (FB2) were kindly provided by Beijing Academy of Agriculture and Forestry (Beijing, China). Hydrofluoric acid (HF), hydrogen peroxide (H_2_O_2_), Tween 80, zearalenone (ZEN), glutaraldehyde (GA), sodium dodecyl sulfate (SDS), sodium borohydride (NaBH_4_), and 3-aminopropyltriethoxysilane (APTS) were purchased from Sigma-Aldrich (St. Louis, MO, USA). Corn flour was purchased from Beijing Zhonghua Shilian Trade Centre (Beijing, China). The optical fiber regeneration buffer was bought from Beijing Hanyue Rujia Biotech. (HY-4-003, Beijing, China).

### 2.2. Instrumentation

A EWOF sensor was bought from Beijing Reliance S&T Co. (Beijing, China) and its basic components were described in the literature [34]. Optical fibers (UV 576/600) were purchased from Beijing Scitlion Technology Co., Ltd. (Beijing, China). Spectrophotometer (UV-2550) was purchased from SHIMADZU CO., LTD. (Tokyo, Japan). Vortex (Vortex-Genie 2) was purchased from Scientific Industries (America). High-speed temperature-controlled centrifuge (3-30K) was purchased from Sigma (Germany). Electronic balance (CP224S SARTORIUS AG) was brought from Shjingmi. Co., Ltd. (Shanghai, China). PH meter was purchased from ShengCi Co., Ltd. (Shanghai, China). Oven (DHG-9055A) was purchased from Shanghai Hengyi Co., Ltd. (Shanghai, China). Ultrasonic cleaner (KH-4000KDE) was purchased from Kunshan Hechuang Co., Ltd. (Shanghai, China).

### 2.3. Functionalization of the Optical Fiber

The modification method of the optical fiber is the same as that reported previously by our group [34,35,37]. Briefly, a razor blade was used to strip 3.5 cm of the resin layer at one end of the fiber. The fiber was immersed vertically in HF until the exposed portion of the fiber etched into a tapered structure with a diameter of approximately 230 µm. After scraping off the remaining cladding on the upper end of the fiber, the fiber was cleaned with a freshly prepared piranha (H_2_SO_4_/H_2_O_2_ = 3:1 *v*/*v*) solution at 120 °C, then dried at 70 °C. The dried fiber was completely immersed in a toluene solution containing 2% (*v*/*v*) APTS for one hour at room temperature. The fiber was then completely immersed in a solution containing 2.5% (*v*/*v*) glutaraldehyde and incubated at room temperature for 3 h to introduce aldehyde groups on the surface of the fiber. Afterwards, the aldehyde-based fiber was immersed in 650 μL of 500 nM amino-modified DNA solution and incubated at room temperature for 7 h to covalently immobilize the aptamer on the fiber. The fiber was then immersed in a 0.3% (*m*/*v*) sodium borohydride solution for half an hour at room temperature. Before detection, the optical fiber was immersed in a 1% (*m*/*v*) aqueous solution of Tween 80 and allowed to stand for half an hour to complete the sealing of the optical fiber interface. We also fabricated the aptamer-functionalized optical fiber without the Tween 80 layer for comparison.

### 2.4. Corn Flour Sample Pretreatment

Two grams of corn flour was mixed with 10 mL of 50% acetonitrile aqueous solution and extracted with shaking for 30 min, and then assisted by ultrasound for 20 min. The mixture was centrifuged (10,000 r/min) for 15 min and the supernatant (extract) was collected. To 90 μL of the extract was added 10 μL of different concentrations of ZEN (50 fM–5 μM, 10-fold gradient).

### 2.5. Detection of ZEN by EWOF Aptasensor

A series of ZEN standard solutions at various concentrations were prepared in the binding buffer. The standard solutions of the corn meal extracts spiked with ZEN as prepared above were mixed with cDNA, respectively. The final sample used for detection is consisted of 1× binding buffer, 30 nM cDNA, and ZEN at different concentrations. The sample was then pumped into the chamber containing the functionalized fiber and held in the chamber for 180 s after flushing the tubing and chamber through binding buffer for 30 s. The sensor response curve was in situ recorded. After each measurement, the sensor was regenerated by rinsing with the regeneration buffer for 2 min. The specificity tests were performed with 100 pM concentrations of various non-target mycotoxins (AFB1, AFB2, DON, AFG1, AFG2, AFM1, OTA, FB1, and FB2) using the same procedure.

## 3. Results and Discussion

### 3.1. The Setup of NADL-EWOF Sensor 

**Figure 1:**
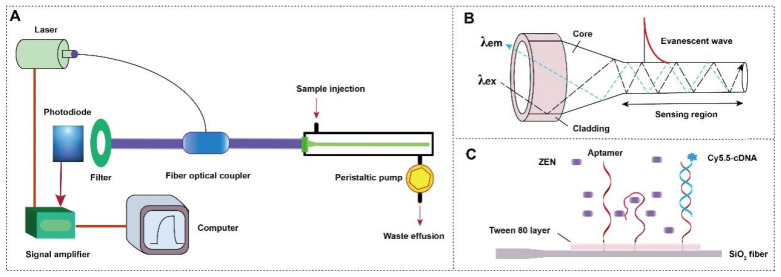
(**A**) The components of a typical EWOF sensor; (**B**) the structure of the tapered fiber and the evanescent wave generated vertical to the fiber surface; and (**C**) the working principle of NADL-EWOF.

The typical EWOF consists of the optical system (laser, optical fiber coupler, filter, photodiode, and signal amplifier), the mechanic fluidic system (peristatic pump, tubes, reaction chamber), and data analysis system (software and computer) (Figure 1A). The tapered optical fiber is installed inside the reaction chamber. The inlet and outlet tubes are connected to the chamber for automatic sample injection and waste elution with controlled flow rate and time. 

The tapered silica dioxide optical fiber was used for this study due to its easy fabrication and low cost (Figure 1B). The total reflection of the incident laser inside the fiber leads to the formation of the evanescent wave propagated vertical to the surface of the fiber. The intensity of the evanescent wave exponentially decreases as the function of the distance away from the fiber surface with the effective length about 100 nm (referred as evanescent wave field). The evanescent wave can excite the fluorescence emission of Cy5.5 inside the evanescent wave field. The intensity of the emission fluorescence is then measured by the photodiode detector after the filter. 

The sensing mechanism of the NADL-EWOF aptasensor for the detection of ZEN is depicted in Figure 1C. The NADL consists of an aptamer layer and a microextraction layer. The physically absorbed Tween 80 layer is used as the microextraction layer. Upon the injection of sample, the ZEN is in situ enriched and purified due to the attraction from the Tween 80 layer and the aptamer. The enriched ZEN competes with the Cy5.5-cDNA to bind with the ZEN-binding aptamer immobilized on the fiber, thereby enabling ultrasensitive signal-off detection of ZEN. 

### 3.2. The Functionalization of Optical Fiber

**Figure 2:**
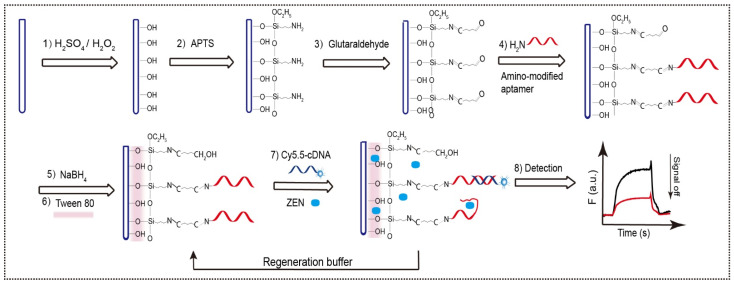
The fabrication and detection process of NADL-EWOF including (**1**) oxidation by piranha, (**2**) salinization, (**3**) linker-coupling to transform the amino group into aldehyde groups, (**4**) coupling of aptamer, (**5**) reduction by NaBH_4_, (**6**) coating with Tween 80, (**7**) injection of the mixture containing Cy5.5-cDNA and ZEN, and (**8**) detection and data analysis.

The optical fiber was functionalized with the ZEN-binding aptamer by following the well-established protocol (Figure 2). Specifically, the amino group modified aptamer 8Z31 was covalently immobilized on the fiber via six steps. (1) The hydroxyl groups on the fiber were generated by oxidation in piranha. (2) The hydroxyl groups were converted into the amino groups by salinization. (3) The amino groups were further transformed into the aldehyde groups by the mild cross-linking reaction between the amino group and one of the two aldehyde groups of glutaraldehyde. (4) The left aldehyde groups then reacted with the amino groups of the aptamer, leading to the covalent immobilization of aptamers on the fiber via the formation of the imine bonds. (5) The less stable imine groups were reduced by NaBH_4_ to form the stable C-N bonds, along with the reduction in the aldehyde groups into the hydroxyl groups. (6) The fiber was further coated with Tween 80 by physical absorption.

The 17-nt Cy5.5-cDNA was complementary to the 3′ end of 8Z_31_ aptamer, which was extending away from the fiber surface. It has been reported that the hybridization at the upper locations is kinetically much more favorable than that near the surface. The Cy5.5 group was modified at the 3′ end of the cDNA to make the Cy5.5 closer to the fiber surface and therefore to generate the higher fluorescence intensity upon hybridization. Upon the injection of sample, ZEN is in situ enriched and purified due to the attraction from the Tween 80 layer and the aptamer. The enriched ZEN competes with the Cy5.5-cDNA to bind with the ZEN-binding aptamer immobilized on the fiber, thereby enabling ultrasensitive signal-off detection of ZEN. We also fabricated the aptamer-functionalized optical fiber without the Tween 80 layer for comparison. Both the Cy5.5-cDNA and the bound and enriched ZEN on the fiber were easily washed off by rinsing with the regeneration buffer, enabling the reuse of the aptasensor.

### 3.3. The Feasibility Test

**Figure 3:**
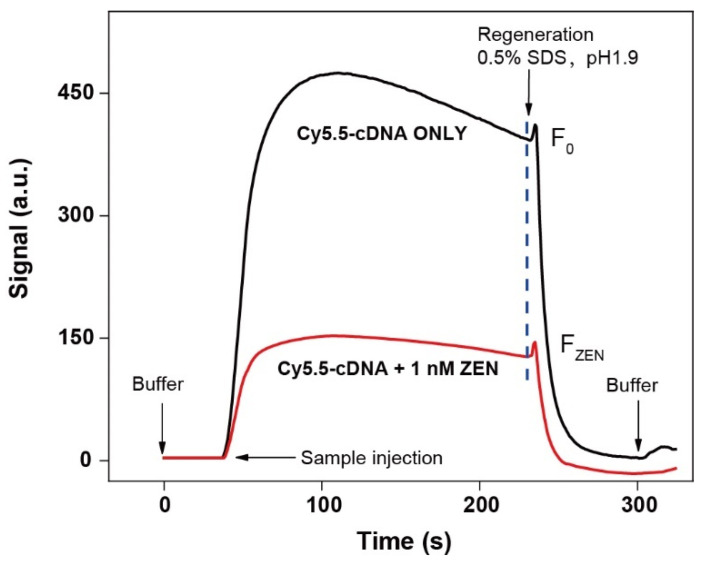
Representative in situ signal responses of the EWOF aptasensor for the detection of 1 nM ZEN (red curve). The black curve is the reference signal in the absence of ZEN (blank sample). The fluorescence intensities of the samples with or without ZEN (F_ZEN_ or F_0_) immediately before the injection of the regeneration buffer (0.5% SDS, pH 1.9) was used to calculate the signal decrease percentage (S% = (F_0_ − F_ZEN_)/F_0_).

We conducted the proof-of-concept experiment using the Tween 80-coated N ADL-EWOF by the respective injection of the blank sample with or without 1 nM ZEN. The fluorescence signal rapidly increases upon the injection of the blank sample (black curve, Figure 3), suggesting that the immobilized ZEN-binding aptamer was able to successfully hybridize with the Cy5.5-cDNA. The maximum signal was reached after the injection of the blank sample, followed by a slight decrease that was probably due to the weak dissociation of cDNA. The signal drops back to the baseline after the injection of the regeneration buffer (0.5% SDS, pH 1.9), suggesting the success of the complete dissociation of cDNA from the fiber surface. 

In contrast, the fluorescence signal increase was substantially decreased when the sample contains 1 nM ZEN (red curve, Figure 3). The result was in good agreement with the proposed competitive sensing mechanism (Figure 2) and therefore proofed the success of the sensor design. The calculated signal decrease percentage (S% = (F_0_ − F_ZEN_)/F_0_) was 65%. It implied that the sensitivity of our ZEN aptasensor should be quite high since the LODs for most classic EWOF aptasensors were around 1 nM. Upon the injection of the 0.5% SDS solution, the signal also drops back to the baseline, which indicated the complete dissociation of cDNA from the fiber surface. The fiber was further rinsed with the commercial regeneration buffer for 2 min to wash off the bound ZEN. 

### 3.4. Ultrasensitive Detection of ZEN via NADL-EWOF Aptasensor

**Figure 4:**
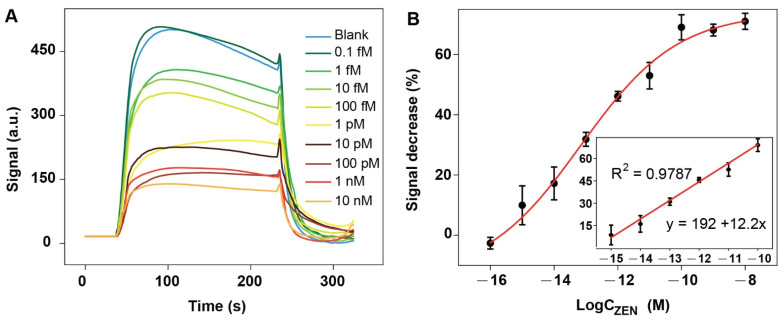
Ultrasensitive detection of ZEN in buffer by NADL-EWOF aptasensor: (**A**) original response curves for the detection of samples containing ZEN at a wide concentration range; (**B**) the titration curve generated according to the signal decrease percentage (S%). The fluorescence intensities used for the calculation of the S% were indicated in (**A**). The error bars in figure (**B**) are the standard deviations derivatized from the three replicates.

The samples containing different concentrations of ZEN were sequentially pumped into the chamber installed with the optical fiber from low to high concentrations of ZEN. The statistically meaningful signal decrease was observed when the concentration of ZEN was 1 fM (Figure 4A). The fluorescence signals were gradually decreased as the increase in the ZEN concentration in the concentration range from 1 fM to 0.1 nM. The signal decreased percentage reached level-off when the concentration of ZEN was 0.1 nM. The LOD (S/N = 3) based on the 3 times standard deviations of the blank sample was 2.31 fM (equivalent to 7.34 × 10^−7^ ng/mL). The semilog linear dynamic range was from 1 fM to 100 pM, which covers five orders of magnitude. Comparing to the reported 8Z_31_-based aptasensors (Table 1), our sensor has the lowest LOD. Our sensor is even more sensitive than most electrochemical aptansensors (SI, Table S2), but much simpler and faster. In addition, neither enzymes nor nanomaterials are required, which lowers the cost and favors the reproducibility as well.

**Figure 5:**
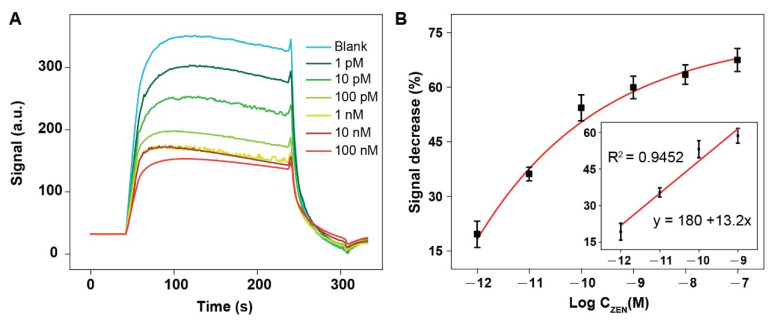
Detection of ZEN in buffer by EWOF aptasensor without Tween 80 coating: (**A**) original response curves for the detection of samples containing ZEN at a wide concentration range; (**B**) the titration curve generated according to the signal decrease percentage (S%). The fluorescence intensities used for the calculation of the S% were indicated in (**A**). The error bars in figure (**B**) are the standard deviations derivatized from the three replicates.

To demonstrate the importance of the Tween 80 layer, we also fabricated the optical fiber without the Tween 80 coating and performed the detection of ZEN using this fiber. The statistically reproducible signal decrease was observed when the concentration of ZEN was 1 pM (Figure 5A). The LOD (S/N = 3) was 2.31 pM. The semilog linear dynamic range was from 1 pM to 1 nM. Comparing to the Tween 80-coated NADL-EWOF aptasensor (Figure 4), the low LOD was about 1000-fold higher, and the linear dynamic range was 100-fold narrower. The results clearly demonstrated the function of Tween 80 for enhancing the sensitivity of the sensor. We also performed the multiple repeated experiments for both aptasensors with and without Tween 80 coating. The reproducibility of the NADL-EWOF aptasensor was excellent, while the EWOF without Tween 80 coating showed limited reproducibility (data not shown). 

### 3.5. Regeneration Performance Tests

**Figure 6:**
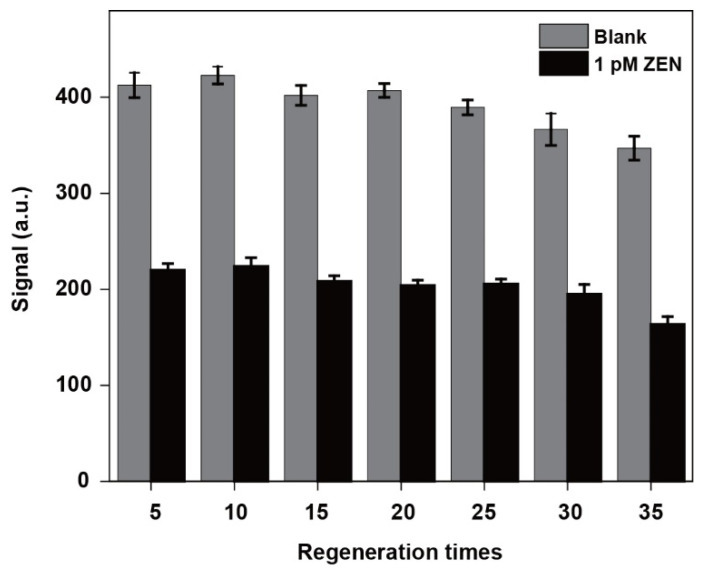
Regeneration tests of the NADL-EWOF aptasensor. The fluorescence intensities were the averaged values of every 5 cycles and background subtracted.

To explore the reusability of the NADL-EWOF aptasensor, we performed reproducibility tests. The 35 cycles of tests were performed, and a test cycle included the sequential injections of the blank sample (cDNA only), 0.5% SDS (pH 1.9), the ZEN sample (1 pM ZEN and cDNA), and the regeneration buffer. The sensor was successfully regenerated for up to 28 cycles (Figure 6) according to the regeneration criteria suggested in the literature [38]. The good reusability of the aptasensor greatly reduces the detection cost. It also improves the reliability of the results since the repeated measurements could be performed using the same fiber. In fact, every few aptasensors can be reused. 

### 3.6. Specificity Tests

**Figure 7:**
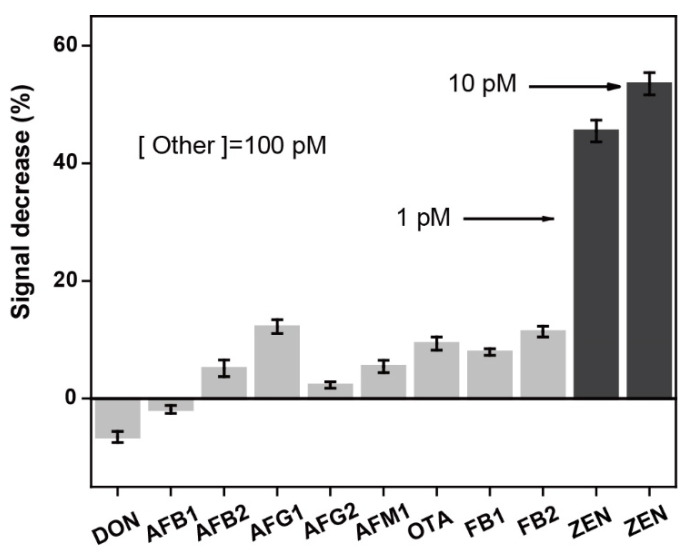
Specificity tests against a panel of common mycotoxins. DON: deoxynivalenol; AFB1: aflatoxin B1; AFB2: aflatoxin B2; AFG1: aflatoxin G1; AFG2: aflatoxin G2; AFM1: aflatoxin M1; OTA: ochratoxin A; FB1: fumonisin B1; and FB2: fumonisin B2. All the measurements were repeated three times and the errors were calculated from the three repeats.

The good reusability of the NADL-EWOF aptasensor allowed us to perform the specificity tests using a single optical fiber, enabling the highly reliable results. We assessed the specificity of the aptasensor against a panel of common mycotoxins including deoxynivalenol (DON), aflatoxin B1 (AFB1), aflatoxin B2 (AFB2), aflatoxin G1 (AFG1), aflatoxin G2 (AFG2), aflatoxin M1 (AFM1), ochratoxin A (OTA), fumonisin B1 (FB1), and fumonisin B2 (FB2). The results showed that our sensor had excellent specificity for ZEN (Figure 7). Specifically, the signal decrease percentages were all less than 13% when the aptasensor was challenged with 100 pM of the other mycotoxins except ZEN. In sharp contrast, the signal decrease percentages in the presence of 10 and 1 pM of ZEN were 54 and 45%. The results indicated that the specificity of the sensor was quite high. 

### 3.7. Real Sample Analysis

**Figure 8:**
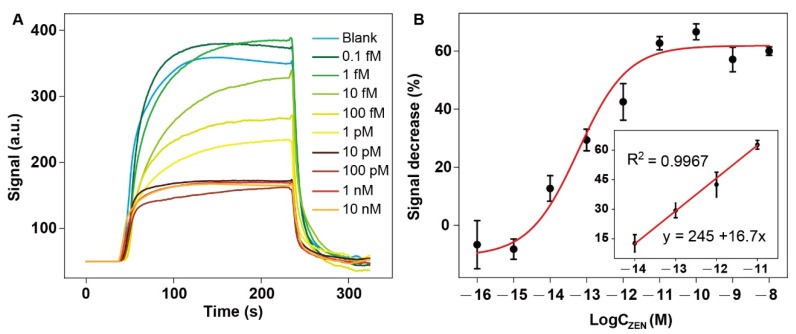
(**A**) The dose–response curves and the calibration curve (**B**) of the NADL-EWOF aptasensor upon the injection of corn extract samples spiked with ZEN at a broad concentration range. The concentrations of ZEN shown in the figures are the final concentrations of ZEN in the 1000-fold diluted corn flour extracts. The actual ZEN concentrations in the undiluted corn flour extracts are 1000-times of the values shown in the figures.

We tested the applicability of the NADL-EWOF aptasensor for the detection of ZEN spiked in the diluted corn flour extract. The fluorescence responses gradually decreased as the increase in the ZEN concentration. The statistically meaningful fluorescence decrease was observed when the concentration of ZEN was 10 fM in 1000-fold diluted corn extract (Figure 8A). The results indicated that the aptasensor was capable for the ultrasensitive detection of ZEN in real sample matrix. The calculated LOD (S/N = 3) according to the fitted calibration equation was 18.4 ± 4.0 fM (corresponding to 18.4 ± 4.0 pM in the undiluted corn flour extracts), which was equivalent to 29.3 ± 6.4 ng/kg and more than 1000-fold lower than the allowed maximum ZEN residue set by China (60 μg/kg) and EU (20 μg/kg). The semilog linear dynamic range was from 10 fM to 10 pM (corresponding to 10 pM to 10 nM in the undiluted corn flour extracts). The above results demonstrated that the NADL-EWOF aptasensor had great potential for the rapid and sensitive detection of ZEN in real samples.

## 4. Conclusions

In conclusion, we successfully constructed the first NADL-EWOF aptasensor for the detection of ZEN. Our sensor possesses the unprecedented sensitivity and represents the most sensitive aptasensor reported so far. Furthermore, it showed the excellent specificity against nine most common mycotoxins. The sensor also has great anti-matrix interference capability, which allows the direct detection of ZEN-spiked in the corn flour extracts simply by sample dilution. In addition, the sensor was successfully regenerated up to 28 times simply by rinsing with the regeneration buffer for 2 min. The total assay time for a single measurement including regeneration and the sample extraction was about 1 h. Our sensor is highly attractive for the practical applications based on its multiple advantages including high sensitivity, good specificity, and strong resistance to matrix interference, rapidity, and low detection cost. The one limitation of NADL-EWOF is its low throughput. The EWOF sensors with multiple detection channels and the plane waveguide sensors are rapidly developed in recent years and can overcome this problem. 

## Figures and Tables

**Table 1:** The 8Z_31_-based aptasensors reported in recent years.

Aptasensor	LOD ^a^ (ng/mL)	Linear Range (ng/mL)	Strength	Weakness	Assay Time ^b^	Cost (USD)	Ref.
Competitive ELAA	0.7	1–10^4^	High throughput	Immobilization of ZEN	3 h	10–20	[24]
Colorimetric lateral flow assay	20	5–200	Convenient	Low sensitivity	5 min	3–5	[25]
AuNP-ELAA	0.08	0.1–160	High throughput	Limited shelf-time of AuNP-aptamer	1 h	-	[26]
Mesoporous SiO_2_-fluorescence	0.012	0.005–150	Simple operation	Long assay time	2.5 h	-	[27]
CoSe_2_/AuNRs -enzymatic- Electrochemical	1.37 × 10^−6^	10^−5^–10	High sensitivity	Complicated nanomaterial and electrode preparation; multistep detection; need of enzyme	6 h	-	[28]
NADL-EWOF	7.34 × 10^−7^	3.18 × 10^−7^–3.18 × 10^−2^	High sensitivity; high anti-matrix interference; low-cost	Low throughput	6 min ^c^	0.5 ^d^	This work

^a^ LOD for the detection of ZEN in buffer; ^b^ excluding the time for sample treatment; ELAA: enzyme-linked aptamer assay; AuNP: gold nanoparticle; ^c^ no sample treatment required; ^d^ cost for one test.

## Data Availability

Data is contained within the article.

## Supplementary Materials

The following supporting information can be downloaded at: https://www.mdpi.com/article/10.3390/bios12070438/s1, Table S1: ZEN-binding aptamers used in the aptasensors; Table S2: Literature reported aptasensors for the detection ZEN. Refs. [39,40,41,42,43,44,45,46,47,48,49,50,51,52,53,54,55,56,57,58,59,60,61,62,63,64,65,66,67,68,69,70,71,72,73,74] are cite in Supplementary Materials file.
