# Supplementary material for: Evanescent Wave Optical-Fiber Aptasensor for Rapid Detection of Zearalenone in Corn with Unprecedented Sensitivity
Source: Biosensors (Basel). 2022 Jun 22;12(7):438. doi: 10.3390/bios12070438 (PMC9313073; doi:10.3390/bios12070438)
Supplement: Supplementary file 1 [file biosensors-12-00438-s001.zip › biosensors-1775658-supplementary.pdf]

Supporting Information

Evanescent Wave Optical-Fiber Aptasensor for Rapid Detection of Zearalenone in Corn with Unprecedented Sensitivity

Haixu Zhao†, Shang Ren†, Zhenzhe Wei, and Xinhui Lou*

Department of Chemistry, Capital Normal University, Xisanhuan North Road. 105, Beijing 100048, China

† The authors contributed equally to this work.

*To whom correspondence should be addressed. Tel: +86-10-68902491 ext. 808; E-mail: xinhuilou@cnu.edu.cn

Table S1. ZEN-binding aptamers used in the aptasensors

Aptamer name	Sequence (5'-3')	Length (nt)	Kd (nM)	Ref.
Z31N	ATGGTACATTACTATCTGTAATGTGATAT	29	11.77 ± 1.44	[1]
8Z31	TCATCTATCTATGGTACATTACTATCTGTAATGTGATATG	40	41±5	[2]
ZEN-62	CTACCAGCTTTGAGGCTCGATCCAGCTTATTCAATTATACCAGCTTATTCAATTATACCAGC	62	15.2±3.4	[3]
ZEN-38	GATGGGGAAAGGGTCCCCCTGGGTTGGAGCATCGGACA	38	2200±400	[4]
Number46	GGAATTCTTGATGTTGCCTGGGATTGTTTGGGCCTTGTGTTTTCTTCCGTTCCAACTTAGTAGGATCCCGAA	72	50.5 ± 5.4	[5]
ZEN-80	AGCAGCACAGAGGTCAGATGTCATCTATCTATGGTACATTACTATCTGTAATGTGATATGCCTATGCGTGCTACCGTGAA	80	-	[6]
Z100	ATACCAGCTTATTCAATTCTACCAGCTTTGAGGCTCGATCCAGCTTATTCAATTATACCAGCTTATTCAATTATACCAGCACAATCGTAATCAGTTAG	98	15.2 ± 3.4	[7]

Note: The aptamer names shown in the Table are either the names used in the reported literatures or named according to the length of the aptamer.

Table S2. Literature reported aptasensors for the detection ZEN

No	Aptasensor	Aptamer probe	LOD (ng/mL)	Dynamic range (ng/mL)	Ref.
1	Colorimetric competitive ELAA	Biotin-$8Z_{31}$	0.7	1-10000	[8]
2	Colorimetric lateral flow assay	HS-$8Z_{31}$-T_{29}	20	5−200	[9]
3	Colorimetric	ZEN-98	4	4−128	[7]
4	Enhanced ELAA	HS–GAT-$8Z_{31}$-Biotin	0.08	0.1–160	[10]
5	Colorimetric-AuNP	ZEN-38	10	10–250	[11]
6	Colorimetric-aptamer walker, Exo III, and AuNPs	HS-ZEN-80	0.01	0.02−80	[12]
7	Fluorescence quenching	ZEN-80-FAM	0.5	0.5- 64	[6]
8	AIR-SE combined with SPR	HS–T_{10}-$8Z_{31}$	0.08	0.01–1000	[13]
9	Green ELAA	Biotin-$8Z_{31}$	0.377	-	[14]
10	Colorimetric-porous platinum nanotubes, p-PtNTs/AuNPs and thionine (Thi) labelled graphene oxide (GO)	HS-ZEN-98	1.7×10^{-4}	5×10^{-4} −500	[15]
11	Fluorescent sensing-Genefinder	$8Z_{31}$	0.1	0.1-200	[16]
12	Fluorescent sensing-mesoporous silica nanoparticle	$8Z_{31}$-FAM	0.012	0.005–150	[17]
13	SERS	HS-$8Z_{31}$	0.001	0.005 -500	[18]
14	Electrochemical	$8Z_{31}$	0.017	0.01-1000	[19]
15	Competitive electrochemical	ZEN-29	1.5×10^{-3}	0.001-100	[1]
16	Ratiometric fluorescent-CdTe quantum dots (QDs)	$8Z_{31}$ and NH_2-ZEN-80	2.4	10 -200	[20]
17	FRET- dual-color AuNCs	NH_2-$8Z_{31}$	5.3×10^{-4}	0.005−100	[21]
18	Optical fiber- localized SPR	HS-ZEN-80	0.102	1- 480	[22]
19	Nanofibers for dispersive solid phase extraction (dSPE)	HOOC-$8Z_{31}$	1.8×10^{-5}	6×10^{-5}–0.01	[23]

No	Aptasensor	Aptamer probe	LOD (ng/mL)	Dynamic range (ng/mL)	Ref.
20	Magnetic separation and time-resolved fluorescent Ln^{3+} inorganic nanoparticles.	Biotin-$8Z_{31}$	2.1×10^{-4}	0.001–10	24
21	Ratiometric fluorescence	$8Z_{31}$-NH_2	3.2×10^{-4}	3.2×10^{-4} -0.32	25
22	Electrochemical-DNA assembly	AAA-$8Z_{31}$-SH	$5.1\ \times10^{-7}$	5.0×10^{-6}-50	26
23	Fluorescence-upconverting nanoparticles	$8Z_{31}$	0.007	0.05–100	27
24	AuNP-affinity monolithic column	$8Z_{31}$	0.05	0.16-60	28
25	Photoelectrochemical-ZnO-NGQDs	ZEN-72-NH_2	3.3×10^{-5}	1.0×10^{-4}–100	29
26	Fluorometric-lighting-up silver nanocluster	GGTCTATTAACGA AGGTCATTAAC-$8Z_{31}$	3.2×10^{-4}	1.3×10^{-3}-100	30
27	Competitive ELAA	ZEN-72	0.2	0.03−2.5	5
28	Fluorometric-gold nanorods and upconversion nanoparticles	$8Z_{31}$-AAATCATGTC	0.01	0.05–100	31
29	Dual-colored persistent luminescence	HS-$8Z_{31}$	2.2×10^{-4}	0.001−50	32
30	Electrochemical -CS@AB-MWCNTs	$8Z_{31}$-NH_2	3.64×10^{-6}	10^{-5} −1	33
31	Fluorometric-DNA tweezer nanomachine	$8Z_{31}$	0.037	0.05- 50	34
32	Fluorometric	$8Z_{31}$	0.25	0.1-1000	2
33	Fluorometric	Biotin-$8Z_{31}$	5.1×10^{-4}	0.001 - 100	35
34	Electrochemical-3D sakura-shaped Cu@L-Glu combined with palladium-platinum nanoparticle (Pd-PtNPs) to obtain Cu@L-Glu/Pd-PtNPs	NH_2-ZEN-80	4.5×10^{-7}	10^{-6} - 100	36
35	Fluorometric-dual-enzyme	Biotin-ZEN-62	2.13×10^{-4}	10^{-3}–1	3
36	Electrochemical-PEI-MoS2-MWCNTs	$8Z_{31}$-SH	1.7×10^{-4}	5×10^{-4} -50	37

No	Aptasensor	Aptamer probe	LOD (ng/mL)	Dynamic range (ng/mL)	Ref.
37	Colorimetric-metal-organic framework nano-container and trivalent DNAzyme	$8Z_{31}$	3.6×10^{-4}	0.01–100	38
38	SERS-Au@Ag core-shell nanoparticles and gold nanorods	HS-$8Z_{31}$	0.054	0.05 - 500	39
39	Electrochemical-AuNPs (rMoS2-Au)	HS-$8Z_{31}$	5×10^{-4}	1×10^{-3}–10	40
40	Electrochemical-CoSe2/AuNRs and 3D structured DNA-PtNi@Co-MOF	CCTCAGCCATATCACATTAGC TGA-$8Z_{31}$	1.37×10^{-6}	10^{-5}- 10	41
41	Pregnancy test strip-catalytic hairpin assembly	HS-$8Z_{31}$-TTGTTCTTCACATTACAGATAGTAATGTA	0.05	0.05–5,000	42

Note: ELAA: enzyme-linked aptamer assay; AIR-SE: attenuated internal reflection spectroscopic ellipsometry; SPR: surface plasmon resonance; SERS: surface-enhanced Raman scattering;

References

1. Azri, F. A.; Selamat, J.; Sukor, R.; Yusof, N. A.; Raston, N. H. A.; Eissa, S.; Zourob, M.; Chinnappan, R., Determination of minimal sequence for zearalenone aptamer by computational docking and application on an indirect competitive electrochemical aptasensor. *Anal. Bioanal. Chem.* **2021,** *413* (15), 3861-3872.
2. Chen, X.; Huang, Y.; Duan, N.; Wu, S.; Ma, X.; Xia, Y.; Zhu, C.; Jiang, Y.; Wang, Z., Selection and identification of ssDNA aptamers recognizing zearalenone. *Anal. Bioanal. Chem.* **2013,** *405* (20), 6573-6581.
3. Yao, X.; Yang, Q.; Wang, Y.; Bi, C.; Du, H.; Wu, W., Dual-enzyme-based signal-amplified aptasensor for zearalenone detection by using CRISPR-Cas12a and Nt.AlwI. *Foods* **2022,** *11* (3), 487.
4. Le, L. C.; Cruz-aguado, J. A.; Penner, G. A., DNA ligand For aflatoxin and zearalenone. *U.S. WO 2011/020198 A1* **2011**.
5. Wang, Y.-K.; Zou, Q.; Sun, J.-H.; Wang, H.-a.; Sun, X.; Chen, Z.-F.; Yan, Y.-X., Screening of single-stranded DNA (ssDNA) aptamers against a zearalenone monoclonal antibody and development of a ssDNA-based enzyme-linked oligonucleotide assay for determination of zearalenone in corn. *J. Agric. Food Chem.* **2015,** *63* (1), 136-141.
6. Goud, K. Y.; Hayat, A.; Satyanarayana, M.; Kumar, V. S.; Catanante, G.; Gobi, K. V.; Marty, J. L., Aptamer-based zearalenone assay based on the use of a fluorescein label and a functional graphene oxide as a quencher. *Microchim. Acta* **2017,** *184* (11), 4401-4408.
7. Zhang, Y.; Lu, T.; Wang, Y.; Diao, C.; Zhou, Y.; Zhou, L.; Chen, H., Selection of a DNA aptamer against zearalenone and docking analysis for highly sensitive rapid visual detection with label-free aptasensor. *J. Agric. Food Chem.* **2018,** *66* (45), 12102-12110.
8. Wei, L.; Tan, G.; Yue, L.; Wu, S.; Wang, Z., Visual detection of zearalenone based on competitive emzyme-linked aptamer assay. *Food Sci. Biotechnol.* **2021,** *40* (4), 76-81.
9. Wu, S.; Liu, L.; Duan, N.; Li, Q.; Zhou, Y.; Wang, Z., Aptamer-based lateral flow test strip for rapid detection of zearalenone in corn samples. *J. Agric. Food Chem.* **2018,** *66* (8), 1949-1954.
10. Sun, S.; Xie, Y., An enhanced enzyme-linked aptamer assay for the detection of zearalenone based on gold nanoparticles. *Anal. Methods* **2021,** *13* (10), 1255-1260.
11. Sun, S.; Zhao, R.; Feng, S.; Xie, Y., Colorimetric zearalenone assay based on the use of an aptamer and of gold nanoparticles with peroxidase-like activity. *Microchim. Acta* **2018,** *185* (12), 535.
12. Taghdisi, S. M.; Danesh, N. M.; Ramezani, M.; Emrani, A. S.; Abnous, K., Novel colorimetric aptasensor for zearalenone detection based on nontarget-induced aptamer walker, gold nanoparticles, and exonuclease-assisted recycling amplification. *ACS Appl. Mater. Interfaces* **2018,** *10* (15), 12504-12509.
13. Caglayan, M. O.; Ustundag, Z., Detection of zearalenone in an aptamer assay using attenuated internal reflection ellipsometry and it's cereal sample applications. *Food Chem. Toxicol.* **2020,** *136*, 111081.
14. Xing, K.-Y.; Peng, J.; Shan, S.; Liu, D.-F.; Huang, Y.-N.; Lai, W.-H., Green enzyme-linked immunosorbent assay based on the single-stranded binding protein-assisted aptamer for the detection of mycotoxin. *Anal. Chem.* **2020,** *92* (12), 8422-8426.
15. He, B.; Yan, X., An amperometric zearalenone aptasensor based on signal amplification by using a composite prepared from porous platinum nanotubes, gold nanoparticles and thionine-labelled graphene oxide. *Microchim. Acta* **2019,** *186* (6), 383.

16. Guo, T.; Chen, J.; Zhou, H.; Zhang, Y.; Ma, L., A fluorescent sensing system based on Genefinder for detection of zearalenone. *Food Ferment. Ind* **2021,** *47* (10), 203-206,213.
17. Tan, H.; Guo, T.; Zhou, H.; Dai, H.; Yu, Y.; Zhu, H.; Wang, H.; Fu, Y.; Zhang, Y.; Ma, L., A simple mesoporous silica nanoparticle-based fluorescence aptasensor for the detection of zearalenone in grain and cereal products. *Anal. Bioanal. Chem.* **2020,** *412* (23), 5627-5635.
18. Chen, R.; Sun, Y.; Huo, B.; Mao, Z.; Wang, X.; Li, S.; Lu, R.; Li, S.; Liang, J.; Gao, Z., Development of Fe3O4@Au nanoparticles coupled to Au@Ag core-shell nanoparticles for the sensitive detection of zearalenone. *Anal. Chim. Acta* **2021,** *1180*, 338888.
19. Azri, F. A.; Eissa, S.; Zourob, M.; Chinnappan, R.; Sukor, R.; Yusof, N. A.; Raston, N. H. A.; Alhoshani, A.; Jinap, S., Electrochemical determination of zearalenone using a label-free competitive aptasensor. *Microchim. Acta* **2020,** *187* (5), 266.
20. Tan, X.; Wang, X.; Hao, A.; Liu, Y.; Wang, X.; Chu, T.; Jiang, L.; Yang, Y.; Ming, D., Aptamer-based ratiometric fluorescent nanoprobe for specific and visual detection of zearalenone. *Microchem. J.* **2020,** *157*, 104943.
21. Khan, I. M.; Niazi, S.; Yu, Y.; Mohsin, A.; Mushtaq, B. S.; Iqbal, M. W.; Rehman, A.; Alhtar, W.; Wang, Z., Aptamer induced multicolored AuNCs-WS2 "turn on" FRET nano platform for dual-color simultaneous detection of aflatoxinB(1) and zearalenone. *Anal. Chem.* **2019,** *91* (21), 14085-14092.
22. Lee, B.; Park, J.-H.; Byun, J.-Y.; Kim, J. H.; Kim, M.-G., An optical fiber-based LSPR aptasensor for simple and rapid in-situ detection of ochratoxin A. *Biosens. Bioelectron.* **2018,** *102*, 504-509.
23. Liu, L.; Ma, Y.; Zhang, X.; Yang, X.; Hu, X., A dispersive solid phase extraction adsorbent based on aptamer modified chitosan nanofibers for zearalenone separation in corn, wheat, and beer samples. *Anal. Methods* **2020,** *12* (48), 5852-5860.
24. Niazi, S.; Wang, X.; Pasha, I.; Khan, I. M.; Zhao, S.; Shoaib, M.; Wu, S.; Wang, Z., A novel bioassay based on aptamer-functionalized magnetic nanoparticle for the detection of zearalenone using time resolved-fluorescence NaYF4: Ce/Tb nanoparticles as signal probe. *Talanta* **2018,** *186*, 97-103.
25. Bi, X.; Li, L.; Liu, X.; Luo, L.; Cheng, Z.; Sun, J.; Cai, Z.; Liu, J.; You, T., Inner filter effect-modulated ratiometric fluorescence aptasensor based on competition strategy for zearalenone detection in cereal crops: Using mitoxantrone as quencher of CdTe QDs@SiO2. *Food Chem.* **2021,** *349*,129171.
26. Qu, C.; Xin, L.; Yu, S.; Wei, M., A homogeneous electrochemical aptasensor based on DNA assembly for zearalenone detection. *J. Chin. Chem. Soc.* **2021,** *68* (10), 1998-2005.
27. Wu, Z.; Xu, E.; Chughtai, M. F. J.; Jin, Z.; Irudayaraj, J., Highly sensitive fluorescence sensing of zearalenone using a novel aptasensor based on upconverting nanoparticles. *Food Chem.* **2017,** *230*, 673-680.
28. Xu, J.; Chi, J.; Lin, C.; Lin, X.; Xie, Z., Towards high-efficient online specific discrimination of zearalenone by using gold nanoparticles@aptamer-based affinity monolithic column. *J. Chromatogr. A* **2020,** *1620*, 461026.
29. Luo, L.; Liu, X.; Ma, S.; Li, L.; You, T., Quantification of zearalenone in mildewing cereal crops using an innovative photoelectrochemical aptamer sensing strategy based on ZnO-NGQDs composites. *Food Chem.* **2020,** *322*, 126778.
30. Yin, N.; Yuan, S.; Zhang, M.; Wang, J.; Li, Y.; Peng, Y.; Bai, J.; Ning, B.; Liang, J.; Gao, Z., An aptamer-based fluorometric zearalenone assay using a lighting-up silver nanocluster probe and catalyzed by a hairpin assembly. *Microchim. Acta* **2019,** *186* (12), 765.

31. He, D.; Wu, Z.; Cui, B.; Jin, Z.; Xu, E., A fluorometric method for aptamer-based simultaneous determination of two kinds of the fusarium mycotoxins zearalenone and fumonisin B-1 making use of gold nanorods and upconversion nanoparticles. *Microchim. Acta* **2020,** *187* (4), 254.

32. Jiang, Y.-Y.; Zhao, X.; Chen, L.-J.; Yang, C.; Yin, X.-B.; Yan, X.-P., A dual-colored persistent luminescence nanosensor for simultaneous and autofluorescence-free determination of aflatoxin B-1 and zearalenone. *Talanta* **2021,** *232*, 122395.

33. Mu, Z.; Ma, L.; Wang, J.; Zhou, J.; Yuan, Y.; Bai, L., A target-induced amperometic aptasensor for sensitive zearalenone detection by CS@AB-MWCNTs nanocomposite as enhancers. *Food Chem.* **2021,** *340*, 128128.

34. Chen, R.; Mao, Z.; Lu, R.; Wang, Z.; Hou, Y.; Zhu, W.; Li, S.; Ren, S.; Han, D.; Liang, J.; Gao, Z., Simple and programmed three-dimensional DNA tweezer for simultaneous one-step detection of ochratoxin A and zearalenone. *Spectrochim. Acta A Mol. Biomol.* **2022,** *272*, 120991-120991.

35. Niazi, S.; Khan, I. M.; Yu, Y.; Pasha, I.; Shoaib, M.; Mohsin, A.; Mushtaq, B. S.; Akhtar, W.; Wang, Z., A "turnon" aptasensor for simultaneous and time-resolved fluorometric determination of zearalenone, trichothecenes A and aflatoxin B-1 using WS2 as a quencher. *Microchim. Acta* **2019,** *186* (8), 575.

36. Ji, X.; Yu, C.; Wen, Y.; Chen, J.; Yu, Y.; Zhang, C.; Gao, R.; Mu, X.; He, J., Fabrication of pioneering 3D sakura-shaped metal-organic coordination polymers Cu@L-Glu phenomenal for signal amplification in highly sensitive detection of zearalenone. *Biosens. Bioelectron.* **2019,** *129*, 139-146.

37. Ma, L.; Bai, L.; Zhao, M.; Zhou, J.; Chen, Y.; Mu, Z., An electrochemical aptasensor for highly sensitive detection of zearalenone based on PEI-MoS2-MWCNTs nanocomposite for signal enhancement. *Anal. Chim. Acta* **2019,** *1060*, 71-78.

38. Sun, Y.; Lv, Y.; Qi, S.; Zhang, Y.; Wang, Z., Sensitive colorimetric aptasensor based on stimuli-responsive metal-organic framework nano-container and trivalent DNAzyme for zearalenone determination in food samples. *Food Chem.* **2022,** *371*, 131145.

39. Chen, R.; Li, S.; Sun, Y.; Huo, B.; Xia, Y.; Qin, Y.; Li, S.; Shi, B.; He, D.; Liang, J.; Gao, Z., Surface-enhanced Raman spectroscopy aptasensor for simultaneous determination of ochratoxin A and zearalenone using Au@Ag core-shell nanoparticles and gold nanorods. *Microchim. Acta* **2021,** *188* (8), 281.

40. Han, Z.; Tang, Z.; Jiang, K.; Huang, Q.; Meng, J.; Nie, D.; Zhao, Z., Dual-target electrochemical aptasensor based on co-reduced molybdenum disulfide and Au NPs (rMoS(2)-Au) for multiplex detection of mycotoxins. *Biosens. Bioelectron.* **2020,** *150*, 111894.

41. He, B.; Yan, X., Ultrasensitive electrochemical aptasensor based on CoSe2/AuNRs and 3D structured DNA-PtNi@Co-MOF networks for the detection of zearalenone. *Sens. Actuators, B* **2020,** *306*,127558.

42. Zhong, Z.-T.; Song, L.-B.; He, Y.-F.; Zhang, B.; Chen, W.; Liu, B.; Zhao, Y.-D., Detection of multiple mycotoxins based on catalytic hairpin assembly coupled with pregnancy test strip. *Sens. Actuators, B* **2022,** *350*, 130911.
